# Complete chloroplast genomes of *Camellia pubipetala* Y. Wan et S. Z. Huang and *Camellia debaoensis* R. C. Hu et Y. Q. Liufu

**DOI:** 10.1080/23802359.2021.1937360

**Published:** 2021-07-14

**Authors:** Hewen Zheng, Sujuan Wei

**Affiliations:** a Key Laboratory of Ecology of Rare and Endangered Species and Environmental Protection, Ministry of Education, Guangxi Normal University, Guilin, PR China; b State Key Laboratory of Biocontrol and Guangdong Provincial Key Laboratory of Plant Resources, School of Life Sciences, Sun Yat-sen University, Guangzhou, PR China

**Keywords:** Yellow camellias, plastid genome

## Abstract

*Camellia pubipetala* Y. Wan et S. Z. Huang and *Camellia debaoensis* R. C. Hu et Y. Q. Liufu are two threatened species of yellow camellias. The complete chloroplast genomes of *C*. *pubipetala* and *C. debaoensis* are 156,811 and 156,854 bp, respectively. They both have a typical quadripartite structure. *C. pubipetala* contains 134 genes, including 90 protein-coding genes, 36 transfer RNA (tRNA) genes, and 8 ribosomal RNA (rRNA) genes. *Camellia debaoensis* also possesses 134 different genes, including 90 protein-coding, 36 tRNA, and 8 rRNA genes. Phylogenetic analysis revealed that *C. pubipetala* is closely related to *Camellia huana*. *Camellia debaoensis*, *Camellia liberofilamenta*, and *Camellia mingii* formed a clade with 75% bootstrap values.

*Camellia* (Theaceae) species with yellow flowers are known as yellow camellias. *Camellia pubipetala* Y. Wan et S. Z. Huang and *Camellia debaoensis* R. C. Hu et Y. Q. Liufu are two such species. *Camellia pubipetala* is a valued ornamental plant with a limited distribution in limestone mountains in Longan County, Guangxi Zhuang Autonomous Region, China (Chang and Ren 1998). It is considered an endangered species (Qin et al. 2017). *Camellia debaoensis* is a newly described species, currently documented only from its type locality (Debao County, Guangxi) with a few individuals. It is considered critically endangered (CR) according to the International Union for Conservation of Nature （IUCN） categories and criteria (Hu et al. 2019). In this study, we sequenced and assembled the chloroplast genomes of *C. pubipetala* and *C. debaoensis* to understand their genetic background.

Fresh leaf specimens of these two species were collected from the Longhushan Nature Reserve (106.62°E, 22.95°N) and Debao County (106.16°E, 23.48°N), respectively. Voucher specimens were deposited at the herbarium of Guangxi Institute of Botany (http://ibk.gxib.cn/, Chunrui Lin, chunruilin@tom.com) under the voucher numbers IBK00430875 and IBK00430867. Total genomic DNA was extracted using the Plant Genomic DNA kit (TIANGEN, Beijing, China). Purified DNA was used to generate short-insert of 400 bp paired-end sequencing libraries according to the Illumina standard protocol and then sequenced on an Illumina NovaSeq 6000 platform. Filtered reads were assembled into a preliminary chloroplast genome using NOVOPlasty version 4.2 with k-mer of 39 (Dierckxsens et al. 2017) and annotated using Plastid Genome Annotator with the default settings (Qu et al. 2019). The plastome of *Camellia impressinervis* (NC022461) was selected as a reference.

The complete chloroplast genomes of *C. pubipetala* and *C. debaoensis* are 156,811 and 156,854 bp, respectively. They both have a typical quadripartite structure. *Camellia pubipetala* contains 134 genes, including 90 protein-coding genes, 36 transfer RNA (tRNA) genes, and 8 ribosomal RNA (rRNA) genes. *Camellia debaoensis* also possesses 134 genes, including 90 protein-coding, 36 tRNA, and 8 rRNA genes.

Phylogenetic analysis was performed using the chloroplast genome sequences of 22 *Camellia* taxa including *C. pubipetala* and *C. debaoensis* (Figure 1). *Polyspora axillaris* was selected as an outgroup. GenBank accession numbers of all species used for phylogenomic analysis are provided in Figure 1. The sequences were aligned using MAFFT version 7.402 (Katoh and Standley 2013), and a phylogenetic tree was constructed using RAxML version 8.2.12 (Stamatakis 2014) with the maximum-likelihood method. The GTRCATI substitution model was selected as the best after 100 bootstrap replications with other parameters kept as default. *Camellia pubipetala* was shown to be closely related to *Camellia huana* with 97% bootstrap support. *C. debaoensis*, *Camellia liberofilamenta*, and *Camellia mingii* formed a clade with 75% bootstrap values.

**Figure 1.**
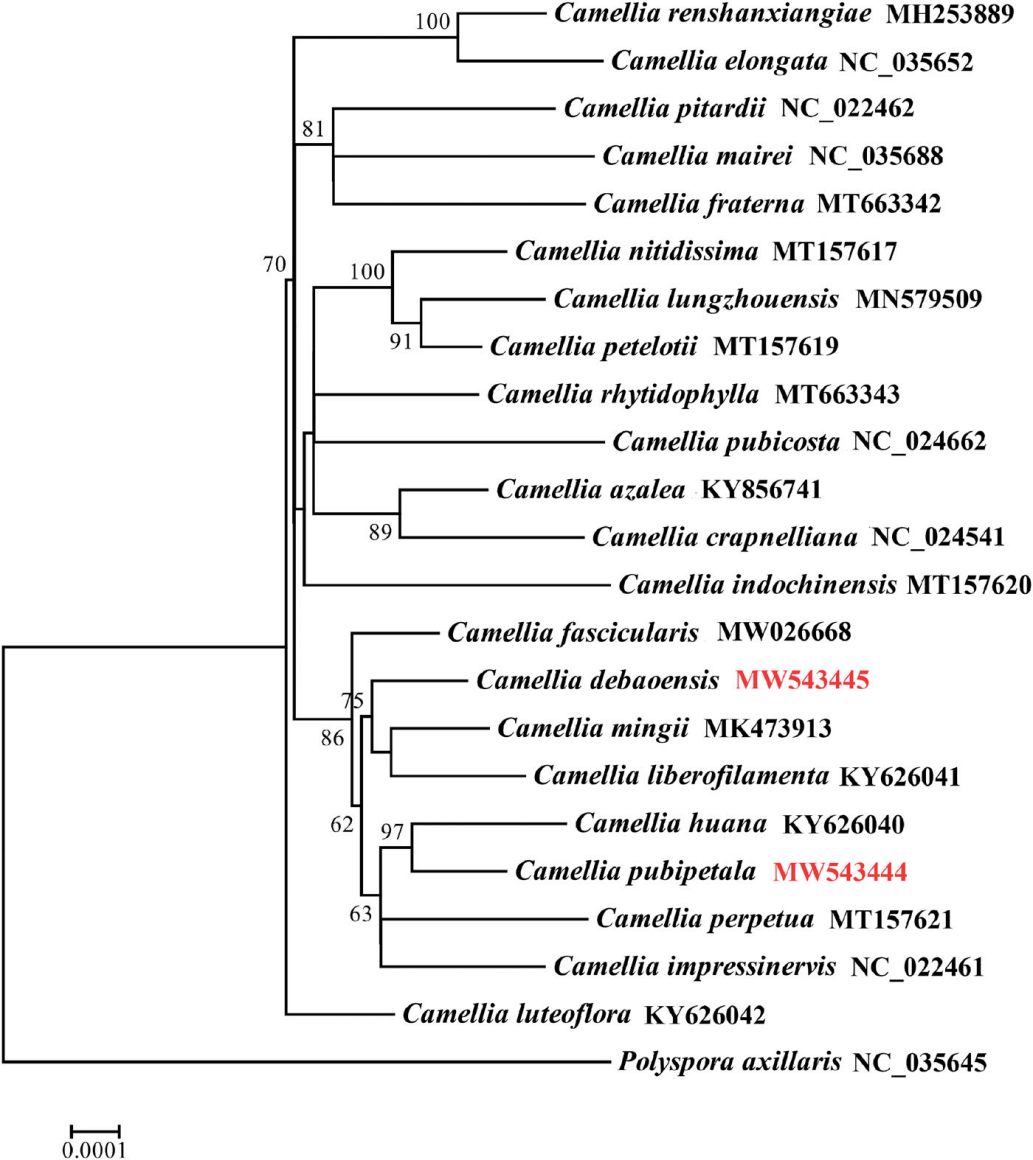
The maximum-likelihood phylogenetic tree was constructed based on 23 complete chloroplast genomes of *Camellia*. *Polyspora axillaris* was selected as an outgroup. Values above branches are maximum parsimony bootstrap percentages.

## Data Availability

The genome sequence data that support the findings of this study are openly available in GenBank of NCBI (https://www.ncbi.nlm.nih.gov/) under the accession numbers MW543444 and MW543445. The associated BioProject, SRA, and Bio-Sample numbers are PRJNA695023, SRR13636538, and SAMN17598212 for *C. pubipetala* and PRJNA695023, SRR13636537, and SAMN17598213 for *C. debaoensis*.
